# Percutaneous Arteriovenous Fistula Creation in a Patient With Recurrent Vascular Access Failures Due to Thrombophilia: A Case Report

**DOI:** 10.7759/cureus.77554

**Published:** 2025-01-16

**Authors:** Hugo Vergara-Pérez, Raúl Diaitz-Usetxi Laplaza, Javier Reque

**Affiliations:** 1 Nephrology Department, General University Hospital of Castellón, Castellón de la Plana, ESP; 2 Radiology Department, General University Hospital of Castellón, Castellón de la Plana, ESP

**Keywords:** chronic kidney disease (ckd), endovascular arteriovenous fistula, hemodialysis, thrombophilia, thrombosis

## Abstract

We present the case of a 62-year-old man who, after experiencing two episodes of thrombosis at the vascular access site for hemodialysis, was diagnosed with antiphospholipid syndrome based on the positivity of lupus anticoagulant on two separate occasions. After initiating anticoagulation therapy, a new arteriovenous fistula was created; however, it encountered another episode of thrombosis. As a result, it was decided to establish a new arteriovenous fistula using a novel endovascular system. Two months after its creation, the fistula met the criteria for physiological maturation, allowing the first hemodialysis session and the removal of the central venous catheter after two months of use.

## Introduction

According to clinical guidelines, a native arteriovenous fistula (AVF) is the preferred choice for vascular access (VA) when initiating hemodialysis (HD), taking precedence over central venous catheters (CVCs) and AVF grafts [1]. This results from a reduced complication rate, lower associated morbidity and mortality, and improved long-term patency [2]. Despite the preference for AVFs as the VA of choice, they can exhibit a high primary failure rate, ranging from 10% to 30%, and can even reach as high as 50% in specific patient groups. Additionally, there is a significant maturation failure rate, with up to 30% of AVFs not maturing adequately within the three months following their creation [3]. Different risk factors for AVF dysfunction have been described, such as obesity, diabetes, gender, and age [4]. Similarly, thrombophilia has been proposed as a possible cause of AVF thrombosis [5].

In 2018, the U.S. Food and Drug Administration (FDA) approved two new endovascular systems for creating native AVFs using minimally invasive methods. Numerous publications have demonstrated the benefits of these systems, showing low complication rates, high patency, and a low rate of reinterventions [6].

We present the case of a male patient with multiple thrombosed AVFs in the context of thrombophilia who underwent a percutaneous AVF as a new option.

## Case presentation

We present the case of a 62-year-old male patient who has been under follow-up by the nephrology team since 2016 due to progressive deterioration of renal function secondary to obstructive uropathy caused by recurrent lithiasis. Despite multiple extracorporeal lithotripsies, there was no improvement in renal function. In June 2017, an ultrasound mapping revealed a cephalic vein at the wrist measuring 2.3 mm and a radial artery of 2 mm. Therefore, in November 2017, a left radiocephalic AVF was created by a surgeon with extensive experience in performing AVFs. Fifteen days later, clinical and ultrasound follow-up by the nephrologists confirmed primary AVF failure due to early, irreparable thrombosis, prompting vascular surgeons to request the creation of a new AVF. After performing a new ultrasound mapping that showed a cephalic vein at the elbow of 3 mm and a brachial artery of 3.1 mm, a new left brachiocephalic AVF was created in March 2018 by a vascular surgeon with over 10 years of experience. Once again, 15 days later, early thrombosis was confirmed. Given the occurrence of thrombosis in two AVFs without an apparent cause, the patient was referred to hematology.

During the consultation, a thrombophilia workup revealed a positive lupus anticoagulant test, while the rest of the study returned negative results. A second lupus anticoagulant test was performed 12 weeks later to rule out false positives, and the result was again positive. Consequently, systemic anticoagulation with acenocoumarol was initiated due to the two episodes of thrombosis and the positive lupus anticoagulant result. After starting anticoagulation, the vascular surgeons requested a new ultrasound mapping, which revealed a cephalic vein of 2.5 mm and a radial artery of 3 mm. Ultimately, a right radiocephalic AVF was created. However, despite anticoagulation, the patient experienced a third episode of early thrombosis. In July 2019, given the necessity to start HD, a CVC was placed in the right jugular vein. Following two years on the HD program utilizing a CVC, the patient was admitted to nephrology due to sepsis secondary to *Staphylococcus aureus*, requiring VA exchange and placement in the left jugular vein.

**Figure 1:**
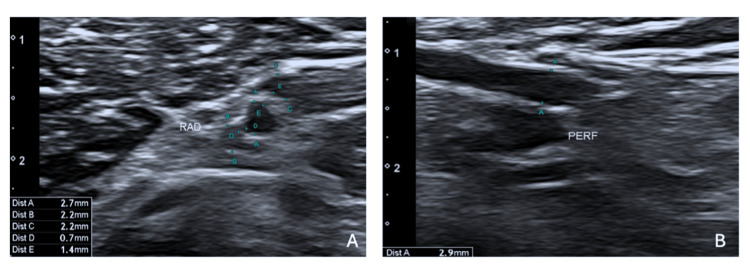
Pre-procedure ultrasound mapping. (A) Radial artery and veins. (B) Perforator vein.

**Figure 2:**
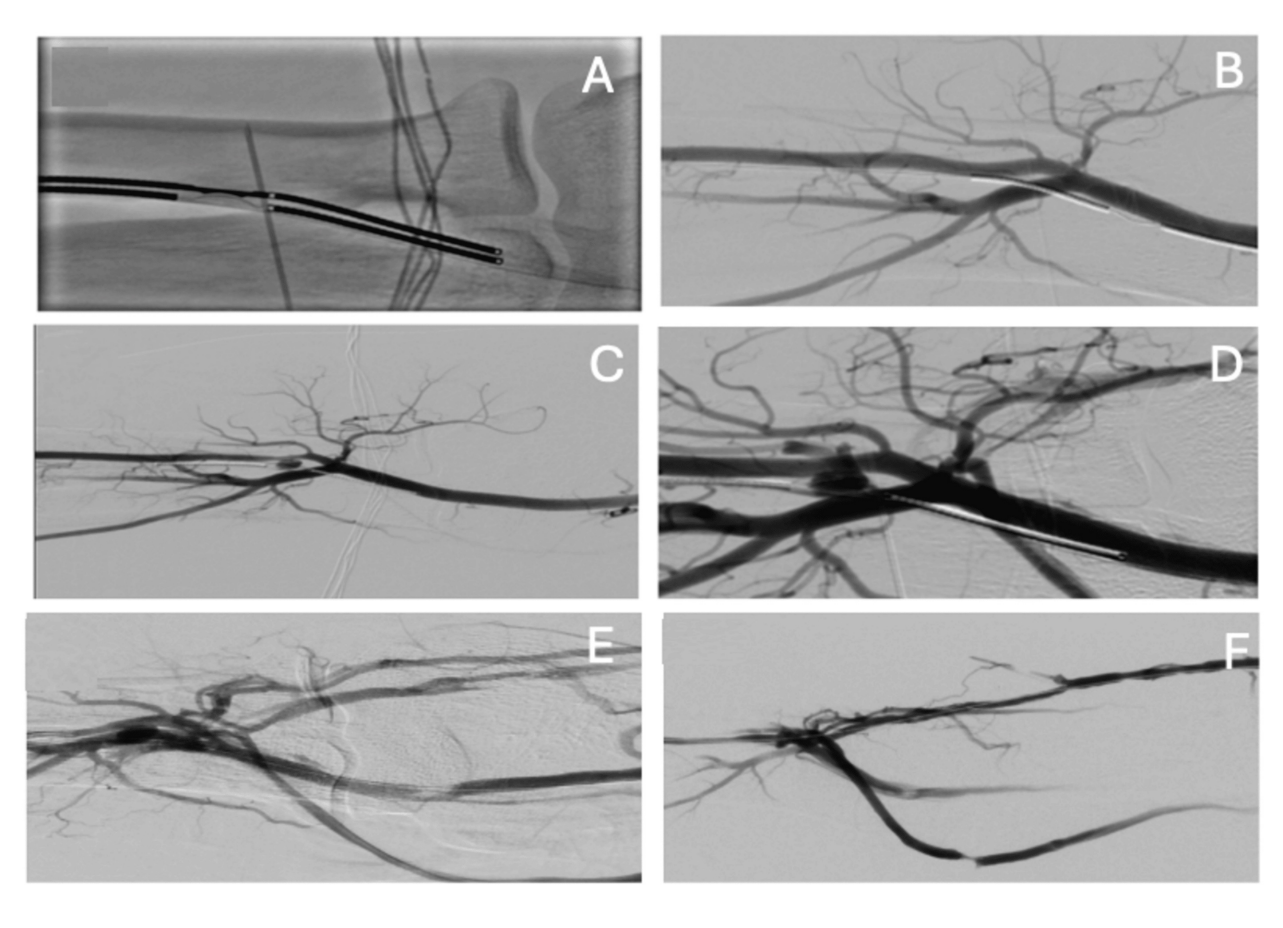
Intraprocedural WavelinQ setup. (A) Aligning the arterial and venous catheters and positioning the radiofrequency electrodes. (B) Pre-creation image of the native vessels with injections through the artery and vein. (C-F) Post-creation image of the EndoAVF.

**Figure 3:**
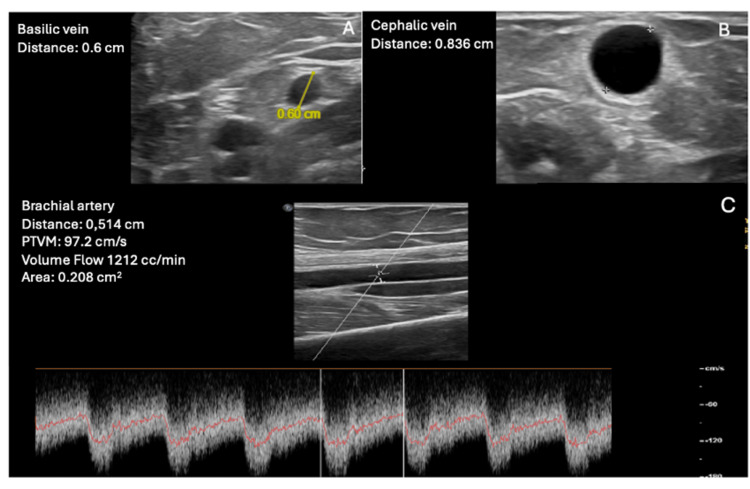
Ultrasound study one month after the procedure. (A) Basilic vein diameter. (B) Cephalic vein diameter. (C) Brachial artery flow.

**Figure 4:**
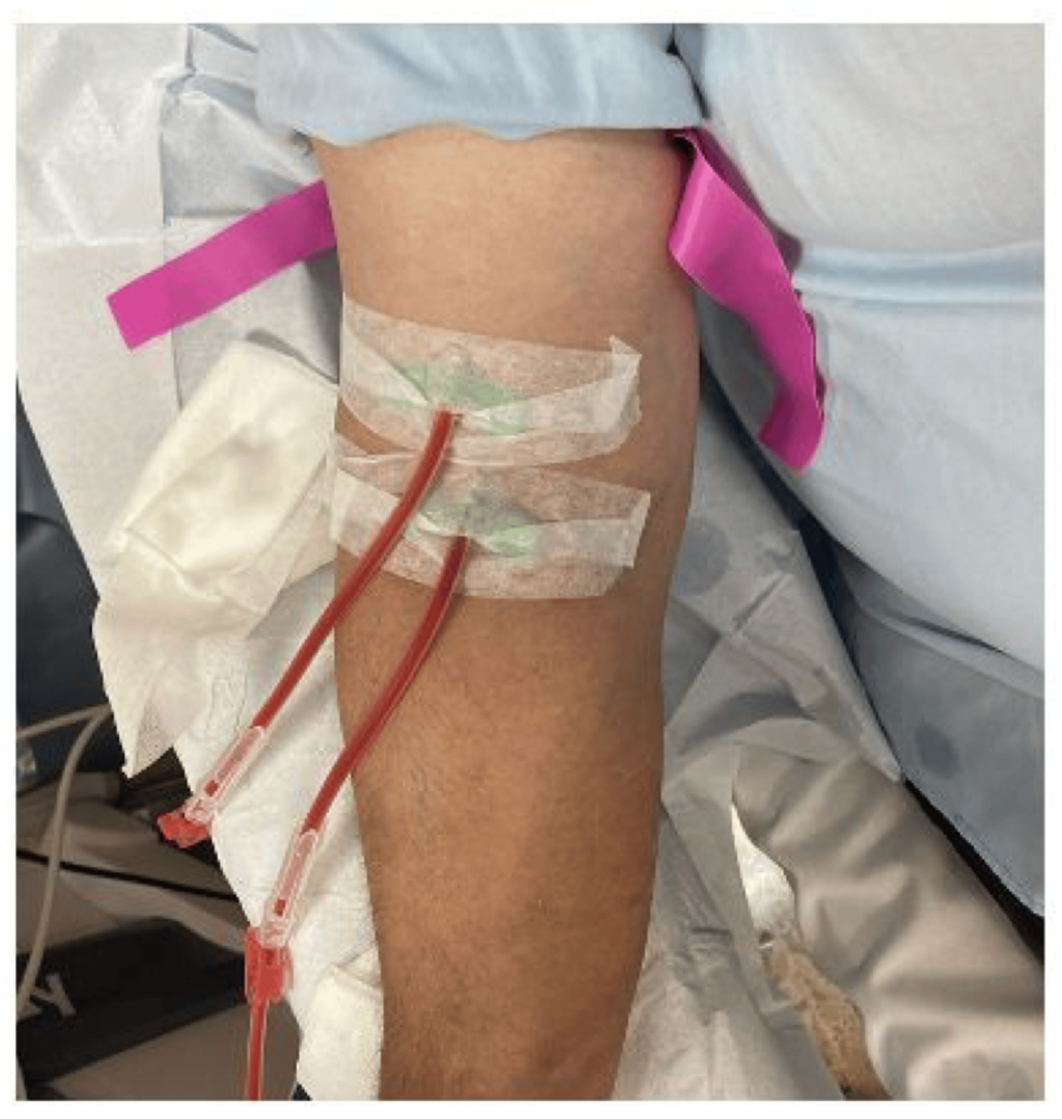
First puncture of the EndoAVF in the cephalic vein pathway, both arterial and venous punctures.

As the patient is relatively young and has a history of multiple AVF issues, a multidisciplinary team discussed the case and contemplated the possibility of performing a percutaneous AVF by interventional radiologists. An ultrasound mapping was conducted to evaluate whether the patient was a candidate for this procedure. The mapping indicated a radial artery of 2.7 mm, radial veins of 2.2 mm, an ulnar artery of 1.6 mm, and ulnar veins of 1 and 0.8 mm, along with a patent perforator vein of 2.9 mm (Figure 1), deeming the patient a good candidate for a right-sided percutaneous AVF. Finally, in June 2024, a percutaneous AVF was created using the WavelinQ™ 4 F EndoAVF system (BD, Franklin Lakes, NJ, US) with a radio-radial approach (Figure 2). An immediate post-procedure measurement of the brachial artery flow indicated 460 mL/min. One month after the procedure, clinical and ultrasound follow-up confirmed proper development of the AVF, with a brachial artery flow of 1,212 mL/min, a cephalic vein diameter of 8.3 cm, and a basilic vein diameter of 6 mm, enabling the first AVF puncture two months later, which confirmed good AVF function with a Qa of 350 mL/min (Figures 3, 4). The CVC was removed after two months of undergoing three HD sessions per week through the AVF.

## Discussion

Although AVFs are the preferred VA due to their higher patency rates and fewer complications, they are also known to have a high primary failure rate, particularly related to early thrombosis and maturation failure. Thrombosis is the most common cause of AVF failure. While many episodes of thrombosis are believed to be linked to anatomical abnormalities such as stenosis, it can also occur in their absence. Various risk factors have been identified that may contribute to early thrombosis of AVFs, including diabetes, being overweight, smoking, advanced age, female gender, the location of the AVF, and small-sized arteries. Other factors that may play a role in early VA thrombosis have been studied, such as polymorphisms of thrombophilic factor genes or the hypercoagulable state observed in patients with end-stage chronic kidney disease. This condition can result from multiple factors, including platelet abnormalities, endothelial factors, the use of erythropoietin, and hyperhomocysteinemia inflammation. Thrombophilia is a hereditary or acquired disorder of hemostasis that increases the risk of thrombotic events. Hereditary thrombophilias include deficiencies of antithrombin, protein C, or protein S and gain-of-function mutations such as factor V Leiden (FVL) and prothrombin gene mutation (PGM). Acquired antiphospholipid syndrome (APS) laboratory features include lupus anticoagulants, anticardiolipin antibodies, and anti-β2-glycoprotein-1 antibodies. Although the reasons remain unclear, studies have indicated a higher prevalence of acquired thrombophilic factors in patients with end-stage renal disease compared to the general population [7]. After experiencing two thrombotic events, our patient underwent a thrombophilia study, which confirmed a diagnosis of APS due to two positive tests for lupus anticoagulant in plasma. Despite some studies not finding an association between VA thrombosis and the presence of antiphospholipid antibodies [8], many others have found a higher risk in these patients [5]. Therefore, initiating anticoagulant treatment with acenocoumarol was decided before creating another AVF. However, despite systemic anticoagulation, our patient experienced a third episode of VA thrombosis. Similar to our patient, a multicenter study conducted by Cervera et al., which included 1,000 patients with APS, demonstrated thrombotic events despite anticoagulation or antiplatelet therapy treatment. The study concluded that new markers and treatments must be identified to improve the prognosis of the disease [9].

Since their approval by the FDA, numerous studies have shown that percutaneous AVFs offer significant benefits in terms of patency while maintaining a low complication rate [6]. Malik et al. demonstrated in a meta-analysis that all studies comparing percutaneous AVFs with surgical AVFs represent an effective and safe alternative for HD patients. Numerous studies have indicated that these systems maintain a very low reintervention rate for VA patency. Although the exact reasons for this are not fully understood, it has been hypothesized that endovascular AVFs may have a lower complication rate due to their minimally invasive nature, potentially resulting in less neointimal hyperplasia and, consequently, a reduced rate of stenosis and thrombosis of the VA [10].

## Conclusions

AVFs for HD can have a high primary failure rate, with thrombosis being the most common cause. Thrombophilia is often underdiagnosed and undertreated in HD patients, potentially contributing to many VA failures. It has been suggested that new minimally invasive systems for creating percutaneous AVFs may result in less neointimal hyperplasia, leading to fewer stenosis and thrombosis in VAs. Consequently, for patients with a history of thrombosis or those at high risk of developing it, these systems could provide a viable option for creating an AVF HD.

## References

[1] Ibeas J, Roca-Tey R, Vallespín J (2017). Spanish clinical guidelines on vascular access for haemodialysis. Nefrologia.

[2] Lok CE, Huber TS, Lee T (2020). KDOQI Clinical Practice Guideline for Vascular Access: 2019 update. Am J Kidney Dis.

[3] Butterly D, Schwab SJ (2002). The case against chronic venous hemodialysis access. J Am Soc Nephrol.

[4] Zhang F, Li J, Yu J (2023). Risk factors for arteriovenous fistula dysfunction in hemodialysis patients: a retrospective study. Sci Rep.

[5] Knoll GA, Wells PS, Young D, Perkins SL, Pilkey RM, Clinch JJ, Rodger MA (2005). Thrombophilia and the risk for hemodialysis vascular access thrombosis. J Am Soc Nephrol.

[6] Vergara-Pérez H, Diaitz-Usetxi Laplaza R, Pérez Alba A (2025). Is endovascular arteriovenous fistula a feasible alternative for hemodialysis patients?. Blood Purif.

[7] Ghisdal L, Broeders N, Wissing KM (2011). Thrombophilic factors in stage V chronic kidney disease patients are largely corrected by renal transplantation. Nephrol Dial Transplant.

[8] Palomo I, Pereira J, Alarcón M, Vasquez M, Pierangeli S (2002). Vascular access thrombosis is not related to presence of antiphospholipid antibodies in patients on chronic hemodialysis. Nephron.

[9] Cervera R, Serrano R, Pons-Estel GJ (2015). Morbidity and mortality in the antiphospholipid syndrome during a 10-year period: a multicentre prospective study of 1000 patients. Ann Rheum Dis.

[10] Li X, Reddy SN, Clark TW, Vance AZ (2023). Endovascular creation of hemodialysis arteriovenous fistulae: the current status and future perspective-a literature review. Cardiovasc Diagn Ther.

